# Process of Posthospital Care Involving Telemedicine Solutions for Patients after Total Hip Arthroplasty

**DOI:** 10.3390/ijerph181910135

**Published:** 2021-09-27

**Authors:** Karolina Kamecka, Anna Rybarczyk-Szwajkowska, Anna Staszewska, Per Engelseth, Remigiusz Kozlowski

**Affiliations:** 1 Department of Management and Logistics in Healthcare, Medical University of Lodz, 90-131 Lodz, Poland; anna.rybarczyk@umed.lodz.pl; 2 Department of Entrepreneurship and Family Business, Social Academy Science, 90-012 Lodz, Poland; astaszewska@san.edu.pl; 3 Narvik Campus, School of Business and Economics, University of Tromsø, 8505 Narvik, Norway; per.engelseth@uit.no; 4 Centre for Security Technologies in Logistics, Faculty of Management, University of Lodz, 90-237 Lodz, Poland; remigiusz.kozlowski@wz.uni.lodz.pl

**Keywords:** process improvement in medicine, posthospital process, posthospital care, patient’s safety, telemedicine solutions, telehealth, total hip arthroplasty

## Abstract

The importance of telemedicine technologies around the world has been growing for many years, and it turned out to be a particularly important issue for conducting some medical procedures during the SARS-CoV-2 pandemic. It is necessary to create interdisciplinary teams to design and implement improved procedures using telemedicine tools. The aim of the article is to develop original, improved posthospital patient care process after total hip arthroplasty (THA) with the use of telemedicine technologies. In the study, a literature review and empirical research were used. The conducted research resulted in the designing an original posthospital patient care process after THA that uses telematics technologies. Due to the use of analyzed telemedicine technologies, the designed patient care process brings a possibility to increase the patient′s safety by monitoring life parameters, allowing for regular, remote contact with specialists and to be supervised remotely. All this may contribute to shortening the convalescence time, reducing the risk of complications, as well as reducing treatment costs. The designed model is ready for further clinical research with the participation of medical staff, patients after THA and patient caregivers.

## 1. Introduction

Telemedicine is a widely prevalent health practice in the medical community, supported by technology-based applications aimed at the efficient delivery of health services, quality of life improvement and providing patients with a wide range of health and socioeconomic benefits [1]. During the COVID-19 pandemic, the use of modern technologies for remote patient–specialist communication, the use of visual technologies, the so-called Smart Wearables and devices belonging to the AT (Assistive Technology) [2] category have experienced a rapid growth [1,3].

The two main grounds for the rapid development of telemedicine technologies are (1) the current epidemic situation and (2) the resulting need for isolation [4,5]. This latter reason involves keeping distance and limitations on interpersonal contacts, the problem of an insufficient number of medical personnel and the generally aging society requiring specialist care. It also includes the next of kin, the support of family members or relatives in the process, supervision or after-surgery convalescence. This study directs attention to cases of patient care after hip arthroplasty (HA). From the healthcare professionals’ and patients’ point of view, e-health solutions may be important in supporting patient care after HA [6,7].

**Figure 1:**
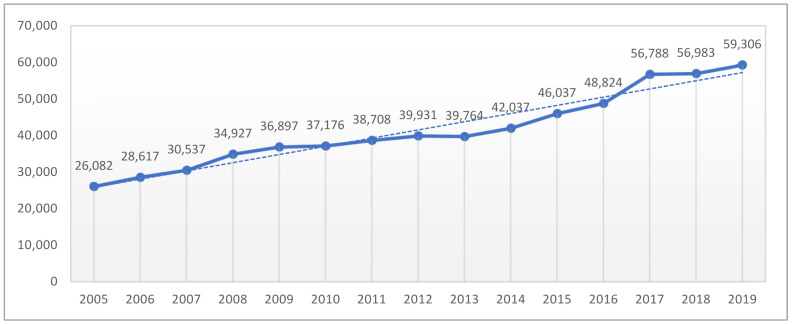
Number of hip arthroplasty procedures conducted in 2005–2019 in Poland according to realization reports from the National Health Fund.

HA is one of the 10 most common surgical procedures in Europe [8] and is the solution for most patients with hip joint disorders causing chronic discomfort or dysfunction of the hip. Since 2005, the realization of HA procedures refunded from the National Health Fund (NHF) are reported in the CBE (Central Base of Endoprotheroplasty). According to data from the CBE, the realization for HA procedures has more than doubled in the period from 2005–2019 (127% increase from 2005 to 2019 year—Figure 1). In absolute numbers, the highest increase in joint arthroplasty is observed in the case of hip joint.

In Poland, hip replacement procedures are performed more often than knee replacements, in comparison to the USA, where an inverse ratio occurs. In the period of 2012 to 2019, there was conducted following number of THA:

- 389,642 THA (63.09% of all joint replacement)—in Poland;
- 625,097 THA (33.3% of all joint replacement)—in USA.

Data show that in both nations the procedure is common and involves a growing trend in the number of conducted procedures [9].

Primary arthroplasty of the hip joint in Poland is shown below [10]:

- In total, in 91% of cases result from primary bilateral coxarthrosis (M16.0), femoral neck fracture (S72.0) or other primary coxarthrosis (M16.1);
- In total, arthroplasty occurs in 87% of cases;
- Women represent over 57% of implants.

In the period from 2005–2019, the number of all arthroplasty procedures in Poland increased as well as the costs of those operations reaching nearly five times higher expenditures. Only in the period from 2014–2019, the amount of EUR 1.31 billion has been spent on two of the most common joint replacement procedures, hip and knee [10] (there is no financial data available in division for type of surgery).

It should be emphasized that this procedure is intended to improve the quality of life of patients [11,12], and this is benchmarked as the treatment of advanced stages of osteoarthritis [13]. Exactly in the view of the effectiveness of reducing pain and improving the functioning of patients, this procedure was announced as the “operation of the century”. Due to the aging of the population and increasing obesity rates, an increased need for joint replacement should be expected [14,15]. In Poland, for a hip replacement procedure, the average age of operated women is 71 years old and 65 years old for men. It is also worth mentioning that the largest share in the total number of operated people were patients aged 60 to 69.

Providing care for patients undergoing HA requires considerable effort beyond the replacement surgery itself to ensure a safe, clinical and cost-effective outcome [16]. Telemedicine enables faster discharge after surgery and contributes to the improvement of rapid procedures without compromising quality, patient safety, functionality, anxiety or other parameters perceived by the patient [17,18]. This system is not intended to replace, but rather to extend, established traditional process of care, while ensuring patient and healthcare provider safety, for example, during a pandemic [19].

These needs can be very different depending on the given technological and organizational environment. In the light of these problems, post-hospital care HA takes on a new dimension and requires a change in the treatment and care process, both by medical professionals (doctors, nurses, physiotherapists) and management staff in medical facilities, as well as the patients themselves and their caregivers. In this process the use of new tools for remote communication (video consultations) or the monitoring of vital parameters and physical activity of patients at home with the use of smart devices and applications is vital [4].

The aim of this article is to develop a project of procedure using telemedicine technologies for posthospital patient care improvement. This article develops a procedure using telemedicine technologies for quality post-hospital care. The studied patients are aged 60 years and older who had undergone total primary HA. We studied more precisely telemedicine applications to support self-rehabilitation at home and its monitoring and minimize the risk of complications.

## 2. Materials and Methods

### 2.1. Research Process Diagram

**Figure 2:**
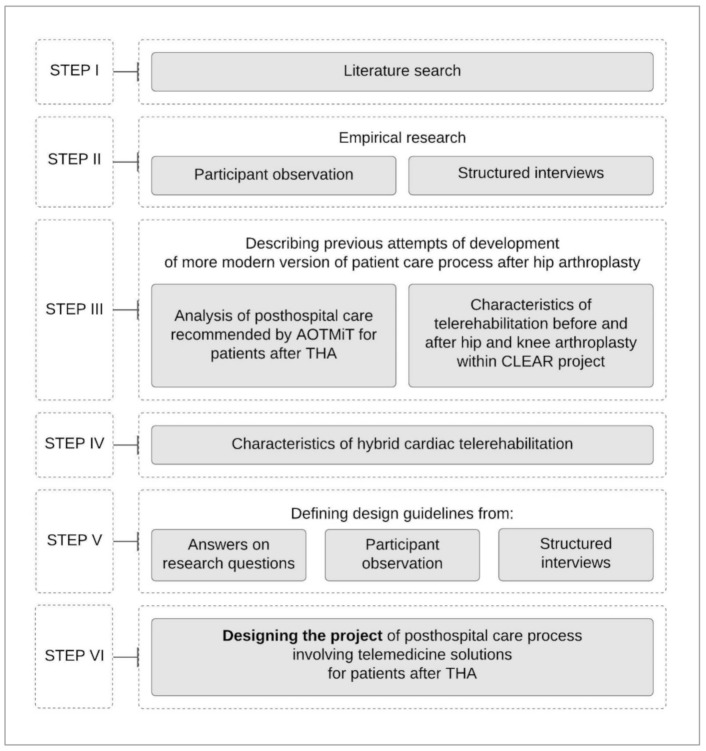
Research process diagram.

In order to clearly present the research carried out, the research process was developed in a graphical form (Figure 2). The 6 steps shown on the left side of the diagram were conducted using methods presented on right side, accordingly.

In the following sections, the individual steps of the research process will be presented, including conclusions/guidelines for designing the Project of improved posthospital patient care process using high technologies.

### 2.2. Literature Search

The published research related to the use of telemedicine technologies in the care of people after total hip arthroplasty (THA) have been reviewed. The following research questions were defined to facilitate the identification of relevant material prior to the commencement of the review work:

(A) What is the process of post-hospital care for patients after THA in Poland and in the world? (B) What telemedicine technologies are used in post-hospital care?
How does the use of telemedicine technologies affect the effectiveness of post-hospital care for patients after THA?
What is the opinion of patients and staff about telemedicine technologies used in post-hospital care after THA?

Based on these questions, a search strategy was built, which consisted in conducting searches in scientific databases—PubMed, EMBASE and Physiotherapy Evidence Database (PEDro), using the search terms: “telemedicine”, “telehealth”, “telerehabilitation”, “ehealth”, “hip arthroplasty”, “postoperative care/methods”, “recovery of function”. The search strategy was as follows: ((“telemedicine” [MeSH Terms] OR “telemedicine” [All Fields] OR “telemedicine s” [All Fields]) AND ((“hip” [MeSH Terms] OR “hip” [All Fields]) AND (“arthroplasty” [MeSH Terms] OR “arthroplasty” [All Fields] OR “arthroplasties” [All Fields]))). 

Expert publications were also searched, databases of projects in which research on improving the care process after HA was carried out. Full-text copies of materials that could potentially meet the inclusion criteria for the review were assessed by the authors against the inclusion criteria. To meet these inclusion criteria and to be eligible for the analysis, the studies had to meet the PICO-based eligibility criteria: The target population (P) was adult patients 18 years of age and older. Intervention (I) was defined as HA. Comparison (C) has been defined as ordinary care after THA (control group) and care after THA with the use of telemonitoring (intervention group). The results (O) included the effectiveness of care and patient and staff satisfaction with the use of telemedicine technologies in post-hospital care after THA. Articles published from January 2005 to March 2021 were included in the review. The search languages were English and Polish. Randomized and non-randomized controlled trials, observational trials and literature reviews were qualified. Administrative documents describing projects to improve health services in people after HA have also been qualified. 

From databases, a total of 102 studies were identified through the literature search: 61 in PubMed, 35 in EMBASE and 6 in the PEDro database. After duplicates, we screened 82 articles; 45 articles met the inclusion criteria.

### 2.3. Data Collection Methods in Our Empirical Research

#### 2.3.1. Participant Observation

Participant observation qualitative study was performed by researcher (KK) in the period of 3 months (August–October 2020). Data were collected at the medical facility specializing in orthopedic surgery including THA. The researcher, as an administration employee, observed the orthopedists, physiotherapists and reception personnel participating in their actions. No voice or visual recordings were made, and notes were therefore only made through participant observation sheets (Supplementary Materials). The study only focused on personnel activities and no patient-related information was identified, assessed or recorded. The participant observer was strictly obliged to respect confidentiality and follow ethical restrictions. All personal data of observed personnel were anonymous and confidential. The participant observer did not ask any questions during observation. Four aspects of the patient care process after THA were of interest (Table 1).

#### 2.3.2. Structured Interviews

The researcher (KK) belonging to the authors of this publication conducted a structured interview, the aim of which was:

- to determine the general stages of posthospital care in everyday clinical practice in a Polish hospital specializing in THA and providing outpatient specialist care in the field of trauma and orthopedic surgery;
- to define knowledge on example telemedicine tools possible to be used in patient care processes;
- to define causes and consequences of possible postoperative complications;
- to define disadvantages of the current posthospital care process.

In total, 12 structured interviews were performed personally by the researcher and notes were taken during every interview (November–December 2020). In this type of interview, the respondents were selected on purpose, based on their positions and experience. Interviewed patients were randomly chosen with inclusion criteria (patient after THA, over 70 years old, without medical complications during hospital stay), whom, according to current medical knowledge, could be included in telemedicine care. Structured interviews with open-ended questions distinct for each group (in Supplementary Materials) were conducted with the head of orthopedic hospital ward—1 person (13 questions), outpatient specialist care orthopedists—2 persons (8 questions), orthopedic outpatient specialist care nurses—3 persons (3 questions), rehabilitation department supervisor (physiotherapist)—1 person (10 questions), outpatient specialist care registration supervisor (administration)—1 person (7 questions), orthopedic supply store—1 person (1 question) and patients after THA—3 persons (14 questions). Qualitative data obtained from the interviews, consistent with the purpose of our research work, were used to design the model.

#### 2.3.3. Ethical Considerations

Research was conducted in accordance with the Code of Ethics of the National Center for Research and Development as our interdisciplinary team work within project titled “InterDoktorMen—Building a new quality and effectiveness of education in the formula of doctoral studies for health care managers at the Faculty of Health Sciences of the Medical University of Lodz”, implemented under the Operational Program Knowledge Education Development 20142020, co-financed by the European Social Fund (no. POWR.03.02.00-00-I027/16-00). The Code of Ethics of the National Center for Research and Development (the “Code” or “Code of Ethics”) is a set of the most important values and principles, setting the basic standards of conduct, as well as shaping the organizational culture of the Center and contributing to ensuring the highest quality of tasks performed. The main values on which the Code is based are cooperation, customer focus, trust, professionalism, development, honesty, impartiality, responsibility, involvement and creating a good atmosphere (more information on the National Centre for Research and Development: https://www.gov.pl/web/ncbr-en (accessed on 26 June 2021)).

For the conduction of interviews and participant observation, consent was obtained from the management of a medical facility specializing in endoprothesoplasty. Informed consent forms were also obtained from every interviewed person. 

### 2.4. Previous Attempts to Develop a More Modern Version of Patient Care Process after Hip Arthroplasty

#### 2.4.1. Analysis of Posthospital Care Recommended by AOTMiT in the Report on Comprehensive Patient Care in Hip Arthroplasty

In 2016, The Agency of Health Technology Assessment and Tariff System (AOTMiT) developed the “Comprehensive care—hip arthroplasty” report in response to the request of the Minister of Health of 16 December 2015 regarding the possibility of implementing comprehensive care or other solutions in the Polish health care system that may improve the quality of services provided and affect the health outcomes of patients in terms of Section H services [20]. In accordance with the recommendation of the Minister of Health, it is necessary to develop a mechanism of incentives for the proper performance of arthroplasty, as well as the rational use of expensive medical products (in the case of this medical procedure, the joint implant constitute a high cost of the entire procedure) [20].

The advantage of the model proposed by AOTMiT is the recognition of the principle of comprehensive care for a patient qualified for the procedure. This model has not been implemented in Poland yet. Due to the rapid technological progress in recent months, mainly due to the COVID-19 pandemic, this model, in the opinion of our research team, requires many changes in the scope of the applicability of telemedicine technologies. In Poland, coordinated posthospital care is provided in a very narrow scope and in a few areas of medicine, i.a., in cardiology.

**Figure 3:**
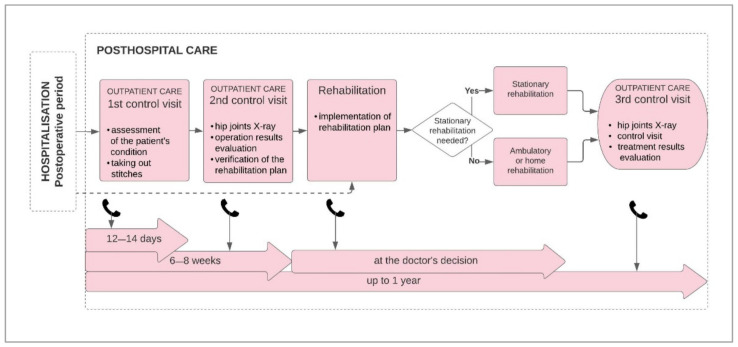
The last stage of the comprehensive patient care model in hip arthroplasty developed on the basis of the AOTMiT report. Source: Own design.

In the model of comprehensive care, the posthospital care period consists of 3 follow-up and rehabilitation visits (Figure 3). Due to the shortages of medical professionals in Poland and the competency model inadequate to the needs of the medical professions, there is insufficient access to health services [21]. Patients after surgery are not covered by sufficient care, which can result in complications, prolonged recovery time or its inappropriate course, generating additional costs of posthospital care. At the same time, according to AOTMiT recommendations, rehabilitation is essential and required for the patient’s full recovery after HA. The AOTMiT process assumes postoperative rehabilitation as part of further hospitalization in the inpatient rehabilitation department in outpatient or home conditions [20]. A noteworthy innovative element of AOTMiT’s model is the introduction of a patient coordinator appointed by the center qualifying for the procedure. The coordinator’s tasks would include arranging appointments, examinations, consultations, performing phone call reminders to the patient about medical appointments, reporting data, as well as collecting data on services provided outside the coordinating center and related to the treatment process of a patient undergoing HA.

#### 2.4.2. Characteristics of Telerehabilitation before and after Hip and Knee Arthroplasty within CLEAR Project

There are various forms of motor telerehabilitation used in orthopedic disorders, many of which are still under development. Some of these are:

- The patient uses at home videos with exercises selected for him by the therapist;
- The patient uses the phone application with exercises selected for him by the therapist;
- The therapist tracks the patient’s exercises on an ongoing basis through video consultation;
- The therapist follows the patient’s exercise performance on an ongoing basis via the internet platform.

The CLEAR research program [22], which was an international project conducted simultaneously in Italy, the Netherlands, Spain and Poland, co-financed by the European Commission, is the chosen benchmark of our research team. The project was aimed at creating a telerehabilitation service and enabling medical professionals (orthopedic doctors and physiotherapists) to design, develop and implement home rehabilitation protocols into clinical practice and support home care in patient physical improvement. The project also aimed to set a European “standard” for home telerehabilitation widely available via internet platform. The project targeted a variety of medical conditions:

- Patients with neurological diseases—research team from Spain;
- Patients with lung diseases and chronic pain—a research team in the Netherlands;
- Patients after a stroke—a research team in Italy;
- Patients with osteoarthritis of the hip and knee joints—research team in Poland.

Patients included in the project received computer equipment and subsequently participated in a rehabilitation cycle lasting 4 weeks at home with individually selected exercises. Patients were trained on how to perform exercises and use the software during their postoperative stay in the hospital ward before discharge from the hospital. The performed exercises were recorded and sent to the physiotherapist for analysis and then video consultations were held, allowing the patient to have visual contact with the physiotherapist and possible correction of the exercise method. The CLEAR project identified the significant value of telerehabilitation, including patients after joint arthroplasty [22]. However, there are currently much more advanced telemedicine technologies, including motion monitoring and visual communication, and this progress should be taken into account in subsequent research projects.

### 2.5. Characteristics of Hybrid Cardiac Telerehabilitation

Cardiac telerehabilitation is one of the first procedures used in remote monitoring and patient care [23,24,25,26]. In Poland, since 2017, hybrid cardiological telerehabilitation (HCTR) has been a service refunded by the NHF. The model of HCTR was developed and implemented at the Stefan Cardinal Wyszynski National Institute of Cardiology, where a project in the field of telerehabilitation in patients with heart failure TELERE-HF was also implemented [27,28,29].

HCTR is one of the most developed among medical procedures using modern telemedicine technologies, both in Poland and in the world. For this reason, it is a good example of a benchmarking solution in relation to other fields of medicine where rehabilitation is an important element of patient care. The team carrying out hybrid cardiac telerehabilitation procedures includes a doctor, physiotherapist, nurse, psychologist, dietitian and medical secretary. Medical equipment (EHO-MINI device, blood pressure monitor, weighing scale) is used for remote monitoring of parameters (ECG, blood pressure, body weight), while telephone contact is used to monitor symptoms. 

The obtained data were sent via the mobile phone network or the internet to a monitoring center equipped with a telemedicine platform enabling the collection, analysis, management and summary of the transmitted data with the possibility of printing and archiving. The model of HCTR consists of two stages [23,30] defined as the preliminary stage (hereinafter Stage I) and the basic stage (hereinafter Stage II). Stage I is short and takes place in hospital (for patients with high cardiac risk) or outpatient (for patients with medium and low cardiological risk) and is aimed to determine the current clinical status, physical capacity of the patient, patient education, planning and conducting several instruction trainings. Stage I consists of:

- An initial visit to the doctor: examination, ECG and exercise test or an ergospirometry test or a six-minute walk test, planning training loads, including the range of the training heart rate and qualification for the appropriate model of HCTR;
- Five days of visits to the outpatient clinic (two to three h each): educational meetings, learning how to use telerehabilitation equipment, learning exercises, dietician’s consultation, psychologist’s consultation, training sessions and lectures on the validity of rehabilitation and a healthy lifestyle.

If Stage I is in the hospital, all examinations and trainings take place during hospitalization, and then the patient, after discharge from the hospital, performs the Stage II of HCTR at home.

For the duration of the Stage II of HCTR, patients receive the following medical equipment: device for monitoring and controlling the training with ECG recording, blood pressure monitor and weighing scale. Stage II usually lasts 6 to 8 weeks and takes place at home. Stage II consist of:

- Between 20 and 24 training sessions (40–60 min each) at home or at the patient’s current location;
- Two procedures repeated daily: permission to start the exercise and training session. Each session is preceded by a phone contact of the patient with the telemonitoring center and data transmission, i.a., resting ECG, blood pressure and body weight measurement. After analyzing the monitoring center, in the absence of contraindications, the patient begins a training session. During each session, telemedicine supervision over the patient is carried out and after the end of the training session, the physiotherapist calls the patient by phone to discuss the course of exercises and determine the degree of effort load;
- A final medical visit to the outpatient clinic: examination, exercise or ergospirometry test or six-minute walk test, evaluation of the effectiveness and summary of the hybrid telerehabilitation cycle and further recommendations.

Hybrid cardiological telerehabilitation is recognized as an effective, safe and well-accepted form of rehabilitation and the transfer of cardiac rehabilitation to the patients’ place of residence makes it possible to increase the availability of rehabilitation programs. The use of telerehabilitation programs and telemetric devices in the Polish health care system could be implemented into orthopedics.

## 3. Results

### 3.1. Design Guidelines

#### 3.1.1. Answers to Research Questions

(A) What is the process of post-hospital care for patients after THA in Poland and in the world? (B) What telemedicine technologies are used in post-hospital care?

The role of eHealth programs to support patients through surgical pathways, including THA, is rapidly growing and offers the potential to improve patient engagement, self-care and outcomes. Remote virtual rehabilitation aroused growing interest in the last decades, and its role has gained importance following the recent spread of COVID19 pandemic [31]. eHealth programs can provide individualized patient care at the preoperative, perioperative and postoperative stages and have the potential to improve patient engagement, self-care and outcomes across the surgical pathway [32].

Currently in Poland, the process of post-hospital care for a patient after HA involves the provision of outpatient specialist care services (personal visits to a specialist doctor in a trauma and orthopedic surgery outpatient care and rehabilitation procedures as part of outpatient physiotherapy) or rehabilitation in a hospital rehabilitation ward. There are no clear guidelines on the frequency of in-person follow-up visits but there are clinical practice guidelines such as:

1. Personal follow-up visits to the orthopedist should take place 6 weeks and 1 year after the procedure, then every 2 years [33].
2. Personal follow-up visits should take place at least in the first year after surgery, 5 years after surgery or earlier if the orthopedic surgeon considers as necessary [34].
3. It is vital to make the patient aware of the importance of personal follow-up visits in the postoperative period. The issue of the frequency of follow-up visits in the postoperative period requires standardization. Clinics schedule 3 control visits in the first year after surgery, at least 3 control visits in the next 10 years and then annual control visits [35].

There are several stages of rehabilitation depending on the period after the procedure [36]:

- First period—immediately after the surgery—including standing upright in the hospital ward, in the absence of complications the patient stays in the hospital ward few days, up to a week;
- Second period—from the end of the week 1 to the beginning of the week 5—including walking on crutches or a walking frame with the relief of the operated limb;
- Third period—from 5 to 12 weeks—including exercises of all muscle groups and improvement of self-service and gradual increase in training load after 4–6 weeks.

According to the recommendations, rehabilitation is required for the full recovery of the patient after HA and is limited by surgical restrictions. Only Westby’s [37] recommendations contain recommendations for post-operative rehabilitation interventions after primary HA. Depending on the functional state of the patient, the presence of comorbidities and postoperative complications, postoperative care may be continued in a hospital setting (in a rehabilitation unit), outpatient clinic or at home (patient’s home, social care facility).

New technologies that have emerged, such as virtual goniometers, wearable sensors (wristbands) and app-based patient questionnaires, have improved clinicians’ abilities to conduct telehealth visits [38]. Digital technology platforms provide a scalable, meaningful approach to engaging patients throughout the continuum of joint replacement care and may serve as a cost-effective adjunct to traditional methods [39]. Some research shows that early postoperative discharge after joint arthroplasty may lead to decreased wound monitoring. A mobile woundcare app with an integrated algorithm to detect complications may lead to improved monitoring and earlier treatment of complications. Scheper and co-authors [40] indicated that introduction of a woundcare app with an alert communication on possible wound problems resulted in a high perceived usefulness and ease of use.

Technological developments combining fitness trackers and tablet use are promising for providing telerehabilitation and for monitoring daily activity [41].

Some challenges still exist, including adaptation of new technologies and widespread accessibility, inability to conduct an in-person orthopedic physical examination and regulatory barriers, such as insurance reimbursement, increased medicolegal risk and privacy and confidentiality concerns. Despite these hurdles, telehealth is here to stay and can be successfully incorporated in any total joint arthroplasty practice with the appropriate adjustments [38]. There is sufficient evidence to recommend the use of telemedical methods in orthopedics [42].

2. How does the use of telemedicine technologies affect the effectiveness of post-hospital care for patients after THA?

In 2014, Sharareh and Schwarzkopf [43] found that patients who had telemedicine visits in the acute postoperative period had less unscheduled clinic visits and calls than those who did not. Furthermore, those patients who used telemedicine post-operatively actually ranked their postoperative satisfaction higher than those who completed in-person visits. In addition, Marsh et al. found that there was not an increased risk of “missing” an acute problem for patients who utilized web-based follow ups [44]. Patients who had telemedicine visits in the acute postoperative period had less unscheduled clinic visits and calls than those who did not. Furthermore, those patients who used telemedicine post-operatively actually ranked their postoperative satisfaction higher than those who completed in-person visits. In addition, Marsh et al. found that there was not an increased risk of “missing” an acute problem for patients who utilized web-based follow up [45]. Telerehabilitation platforms encourage clinician–patient interaction beyond the hospital setting and offers the advantage of cost savings, convenience, at-home monitoring and coordination of care, all of which are geared to improve adherence and overall patient satisfaction [46]. The utilization of an online physician–patient messaging platform can prevent unnecessary visits for normal appearing wounds, while facilitating rapid in-person treatment of wound complications [47].

Some research indicates that remote virtual technologies allow the delivery of high-quality care at reduced costs [18,31,48,49,50,51,52], time and hospital visits reduction [53]. Rosner and co-authors [54] indicated that patient enrollment in digital patient engagement platforms (DPE platform combining remote guidance and telemonitoring) after hip and knee endoprothesoplasty resulted in significant cost reductions, which could be avoided in 90-day hospital admissions, 45.4% nonsignificant relative reduction in 90-day hospital admissions and 54.4% significant relative reduction in 90-day complications. Some scholars have shown that telemedicine reduces costs by reducing the number of medical personnel [55,56,57].

Fernando Dias Correia et al. proved that eHealth programs to support individualized patient education on preoperative preparation, in-patient care and home rehabilitation have the potential to increase patient engagement, enhance patient recovery and reduce potential postoperative complications [58]. The length of postoperative stay was shortened in patients with the TMS solution, without compromising patient-perceived or clinical parameters in patients undergoing elective fast-track surgery. These results indicate that telemedicine can be of value in fast-track treatment of patients undergoing total hip replacement [7,59,60].

Telerehabilitation is a practical alternative to conventional in-person outpatient physical therapy in patients with lower-limb joint replacement [61]. The effect of the three-month telerehabilitation therapy in patients following hip re-placement was equivalent to the usual aftercare taking into account functional testing, quality of life and pain and might be promising addition to already established aftercare process [62]. In Horton et al. [63] study telehealth physical therapy after hip arthroscopy was found to lead to similar outcomes and was cost-effective compared with in-person physical therapy. Other studies indicate the therapeutic usefulness of telerehabilitation systems and tests based on virtual interaction have shown that these can be as effective as traditional treatments [64,65,66]. Telerehabilitation could be delivered by nurses in collaboration with physiotherapists and surgeons as a team. The mobile app is an accessible and flexible delivery medium for telerehabilitation [67].

3. What is the opinion of patients and staff about telemedicine technologies used in post-hospital care after THA?

Several studies describe patient satisfaction with telehealth programs [68,69,70,71,72,73]. Buvik et al. [74] found, in a randomized controlled trial, that 86% of patients having remote consultations preferred a video-assisted consultation for the next visit. In addition, there was no significant difference in patient-reported health after 12 months between the group randomized to receive video-assisted remote consultations and those who received a routine in-person consultation. LeBrun et al.’s [75] study of outpatient arthroplasty telemedicine visits showed high rates of patient satisfaction. Most patients noted decreased costs, with the most common increased comfort and less travel-related anxiety associated following early telemedicine visits. Some research indicates that telerehabilitation programmes can be delivered to patients in their own homes, using readily available technology while maintaining high levels of satisfaction [31,76,77], and it is perceived as enjoyable and engaging and can increase the intensity of rehabilitation [64]. 

In Chen et al.’s study, patients undergoing arthroplasty and their surgeons were satisfied with telemedicine and see a role for its use after the pandemic. The audiovisual quality and the responsiveness of physicians to the concerns of patients determine their satisfaction [78]. In only one study, the use of telemedicine for orthopedic assessments did not result in identifiable differences in patient or surgeon satisfaction compared with in-person assessments [79].

#### 3.1.2. Design Guidelines from Participant Observation

As a result of the participant observation, the following conclusions for the project design were drawn:

1. Types of rehabilitation in posthospital patient care process:
  - ambulatory rehabilitation—stationary;
  - ambulatory rehabilitation—at patient home;
  - rehabilitation ward, recommended depending on the patient’s health condition;
2. Physiotherapists’ instructions directed to patient in hospital in the field of self-rehabilitation at home in posthospital period:
  - oral instructions;
  - presentation of exercise/body and limb movement performance, written instructions (printed instruction with text descriptions and graphic visualization of exercises) given during patients stay at the hospital after THA;
3. Patient registration process for control visits in posthospital period:
  - patients register control visits at outpatient specialist care on the referral received upon discharge from hospital;
  - all information in regard to dates and intervals between visits are given at outpatient specialist care by reception personnel;
  - comprehensive patient care after THA is possible in medical facility offering access to services needed in posthospital patient care (patient can register for control visit at the same day of discharge from hospital at the medical facility);
4. Observation on video consultation tool implemented as commercial service for patients:
  - The orthopedist efficiently uses the video-consultations tool without administration assistance;
  - the video consultation system includes the possibility of electronic medical records storage;
  - the orthopedist using the telemedicine tool has a work schedule previously set up in the telemedicine system;
  - the orthopedist cooperates with administration personnel and shares opinions on the functioning of telemedicine system resulting from his experience after video consultation is conducted.

#### 3.1.3. Design Guidelines from Structured Interviews

As a result of the structured interviews, the following conclusions for project design were drawn:

1. Control visits after THA:
  - control visits are important for monitoring patient condition;
  - there is no strict standardization of the frequency of control visits;
  - number of control visits depends on patient condition;
  - patients do not always attend control visits on the set dates;
  - there is no role of coordinator provided in the structure of the posthospital care process who is the patient’s guardian, e.g., in regard to phone control visits reminders;
2. Rehabilitation after THA:
  - the type of rehabilitation depends on the patient’s health condition;
  - instructed self-rehabilitation at home has an important impact on patient recovery;
  - patients perform self-rehabilitation at home according to the given oral and written instructions with pictures;
3. Common causes of medical complications after THA:
  - lack of patient rehabilitation;
  - patient collapse;
  - failure to follow specialistic instructions (from orthopedist and physiotherapist) regarding temporarily prohibited patient’s body movements;
4. Patient attitude after THA:
  - patient can be stressed/unsure just after surgery in regard to proper body movement, activity and general functioning;
  - patients are open to use orthopedic aids and devices facilitating their functioning;
5. Telemedicine tools:
  - there is no telemedicine tool (telerehabilitation programs, collapse wrist bands) widely known and/or used to supervise patients’ physical condition, activity and the correctness and frequency of exercise at home by the patient;
  - hospital personnel have general knowledge on telemedicine solutions (e.g., hybrid cardiac telerehabilitation was mentioned as application example);
  - patients are open to using collapse sensors if equipped.

#### 3.1.4. Diagram of Current Posthospital Patient Care Process after THA and Synthetic Conclusions for New Process Design 

**Figure 4:**
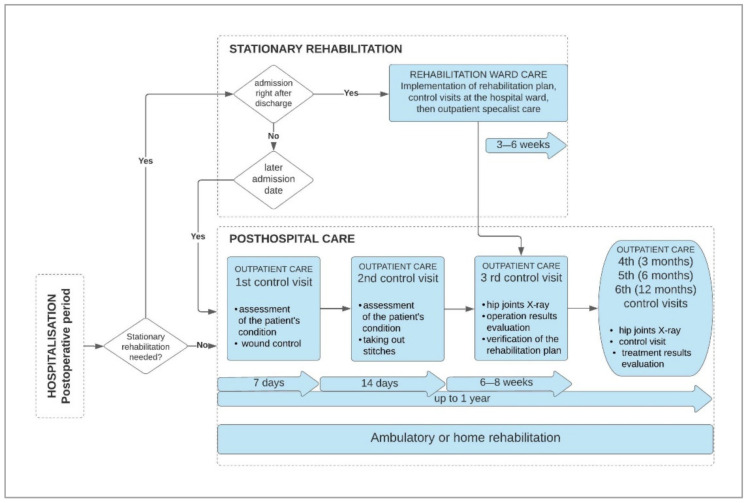
General diagram of the current posthospital patient care process after total hip arthroplasty. Source: own design.

Based on the qualitative data obtained from structured interviews, participant observation and literature review [33,34,35,36,37], the researchers designed a diagram of the current posthospital patient care process after THA (Figure 4). Personal follow-up visits after THA are generally performed in the following order:

- First follow-up visit—takes place one week after the procedure to assess the patient’s general condition and control of the postoperative wound;
- Second follow-up visit—takes place after two weeks to assess the general condition of the patient and remove the stitches;
- Third follow-up visit—takes place after 6–8 weeks to assess the patient’s physical condition, verify the rehabilitation plan and, if necessary, take a control X-ray of the hip joints;
- Fourth, fifth and sixth follow-up visits—take place after 3, 6 and 12 months, respectively, to assess the patient’s physical condition, take a control X-ray of the hip joints, or in individually recommended periods depending on patient condition.

The conducted research procedures provided, in sum, the following synthetic conclusions:

- There is a lack of tools for sufficient patient supervision during his stay at home, i.a., the lack of monitoring of the patient’s collapse, general physical activity in everyday life and realization of rehabilitation program after discharge from the hospital.
- There is a lack of comprehensive care model to ensure proper patient care after THA which is major surgery with risk of complications.

### 3.2. Project of Improved Posthospital Patient Care Process Using High Technologies—Final Results

**Figure 5:**
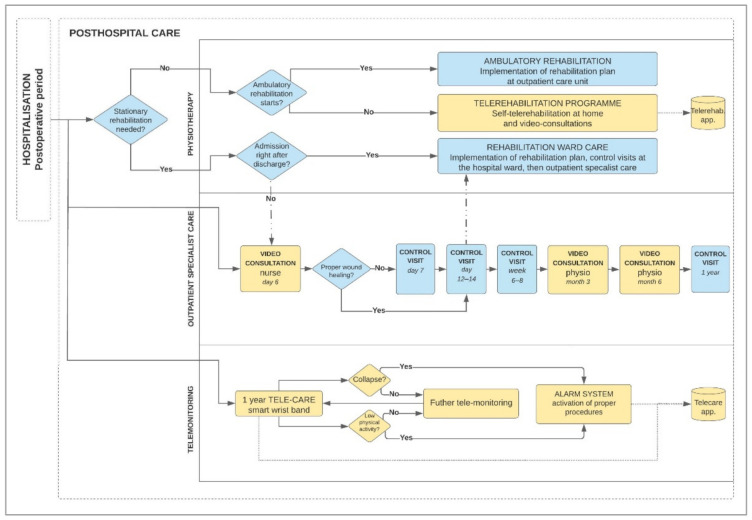
Diagram of posthospital process in patient care at home after hip arthroplasty with telemedicine technologies. Source: Own design.

After analysis of the above-mentioned conclusions, current processes, the AOTMiT model, the CLEAR project and the hybrid cardiological telerehabilitation system, the Project of improved posthospital patient care process using telemedicine technologies was designed. This chapter presents the original proposed process of posthospital patient care after THA using modern visual and wearable technologies (Figure 5). We implemented in the project the most effective and innovative telemedicine tools identified during the research process. The tools implemented in the process may improve posthospital care and affect the sense of security of the patient and his caregivers, as well as reduce the risk of complications, including those resulting from the lack or improper self-rehabilitation at home. 

The process takes into account the process approach (actions and the time in which they should be taken) and functional (human resources and technologies supporting the process) with the use of modern technologies. The project includes the patient’s contact with a doctor, physiotherapist and nurse and has been divided into three interrelated areas:

- Physiotherapy;
- Outpatient specialist care;
- Telemonitoring.

Depending on the medical indications, the patient is referred to the inpatient rehabilitation ward, outpatient physiotherapy or enrolled in the telerehabilitation program at home using visual and wearable technologies, the results of which are monitored by a physiotherapist. In the area of outpatient specialist care, the patient undergoes personal visits and video consultations. After each consultation, both remote and personal, electronic medical records are kept in the medical software of the coordinating center. If the patient is not qualified for hospital rehabilitation or the waiting period for admission to the ward is prolonged, the patient then undergoes the following: first, remote video consultation with a nurse at day 6 for the evaluation of wound healing, monitoring of the general health of the patient in the early postoperative period, verification of the implementation of the recommendations of the attending physician, providing the necessary support in case of additional questions from the patient. This period is fraught with a feeling of uncertainty for patients, e.g., whether they properly perform nursing activities and self-administered anticoagulant injections. Thanks to video consultation, patients can receive professional advice from a qualified nurse. This video consultation determines the necessity of undergoing personal visits at outpatient specialist care in case of problems with wound care or other issues. In the case of problems detection, the patient is immediately referred to, first, personal control visit to an orthopedist at an outpatient specialist care unit (control of wound healing, assessment of the general health of the patient); however, if the wound is healing properly, the patient will be able to come to a control visit with a specialist doctor between 12 and 14 days (removal of the stitches).

Then the following procedures take place: personal control visit to orthopedist after 6–8 weeks (general health assessment, check-up X-rays, verification of the rehabilitation process), video consultation with a physiotherapist after 3 and 6 months (evaluation of the self-rehabilitation process and the rehabilitation process, monitoring and possible correction of the home rehabilitation process, in the case of abnormalities in the course of convalescence and recovery, recommendation of a personal orthopedist or physiotherapeutic control visit, video consultation on these periods are justified due to the expected time of discontinuation of using the elbow crutches between 3–6 months and a long period of lack of contact with orthopedist until the next personal control visit) and a summarizing personal control visit with orthopedist after one year (general health assessment, check-up X-rays, evaluation of the entire rehabilitation and telemonitoring process, including falls and general physical activity).

Assuming that the patient meets the criteria for introducing him to the remote postoperative care system, the patient is educated on the telerehabilitation and telemonitoring program being crucial and necessary element for the proper conduct of remote care before the patient is discharged home. The essence of introducing telecare wristbands integrated with the monitoring center into the posthospital care model should also be emphasized. Owing to the center, not only the data on the patient’s vital parameters are collected on an ongoing basis but also patient’s falls or failure to perform the prescribed exercises. In addition, the patient can report adverse events that may occur during his recovery and/or call for help urgently via telecare wristband. The full period of postoperative care is designed for a period of 1 year. The final number of personal visits would depend on the doctor’s recommendations taking into account the patient’s health condition at following stages of the treatment process.

The software system for a hospital (HIS, Hospital Information System) can be the main database with the possibility of ongoing monitoring. An important role for the system user is the transparency of the visualization of measurements collected from telemedicine devices, applications and systems, which does not require deep independent analysis of individual data. These data would include, i.a., measurement of the patient’s activity, frequency of falls, results of the telerehabilitation program and data from visual technologies, fully enabling a medical interview based on actual data. 

## 4. Discussion

The hospital environment can be a hazardous place due to exposure to possible sources of infection, therefore visits to and time at the hospital should be minimized. Research shows that telerehabilitation programs can be delivered to total hip replacement patients in their own homes, using readily available technology while maintaining high levels of satisfaction. More importantly, telerehabilitation patients appear to achieve non-inferior physical and functional outcomes as those receiving in-person rehabilitation programs [80]. There has been evidence suggesting that real-time virtual rehabilitation may be equivalent to conventional methods for adherence, improvement of function and relief of pain seen in these conditions [46]. The use of technology such as smartphone apps to provide pre-operative education, wearable activity trackers to assist with rehabilitation and the use of telemedicine to complete outpatient appointments may be utilized [81]. We are aware that the telerehabilitation program is a wide area of improvement for current posthospital patient care process in Poland. Currently, the post-hospital care process for a patient after HA and its procedures financed by the NHF in Poland assume the provision of services as part of outpatient specialist care or rehabilitation in a hospital setting at a rehabilitation ward. Rehabilitation is an important component of services financed by the NHF [82], but primarily, telerehabilitation is used in cardiac rehabilitation and to lesser extent in diseases of the musculoskeletal disorder. Identified therapeutic tools such as telerehabilitation systems, applications and wearables are recommended as important elements of comprehensive patient care process development gaining acceptance of use in the medical community and the patient environment. Some studies show that phone conversations bring clear benefits to patients after THA. Hällfors et al.’s [83] research revealed that the service reduced the number of unnecessary visits to the ED (Emergency Department) and worked well in detecting patients requiring follow-up. Electronic patient rehabilitation applications can be used to provide perioperative care at home. According to Davidovitch RI et al. [84], the integration of electronic rehabilitation tools is gaining acceptance within the orthopedic community. In the process designed by our interdisciplinary team, implementation of even more advanced technology than phone conversation service, precisely visual, remote patient–specialist contact via video consultation when a personal visit is not necessary or possible, introduces a strengthening improvement to the patient–specialist communication. Due to this specific type of connection presented in proposed process, both sites can benefit, i.a., the patient has a feeling similar to a personal visit, resulting in an increased sense of security, and the specialist gathers possibility of enriching the interview with non-verbal behavior. 

It should be noted that telehealth is still evolving, and geriatrics is an important specialty considering that elderly people and their careers have unique needs. Therefore, proper patient selection and clear communication paths to solve patients’ problems are key to success. Education of patients [85,86] and medical staff [87] must be part of the implementation of telemedicine technologies in the process of post-hospital care in people undergoing HA. In the process of educational familiarizing with telemedicine technologies all actors of the posthospital care process plays a crucial role in the proposed process. Medical treatment, which is a subject of this research, concerns mainly people over 60 years old for whom limitations and difficulties should be defined and eliminated or minimalized by external support, e.g., patient caregivers from family or closest environment. The acceptance of all technology users in the described process is the basis for the effectiveness of defined innovative technologies implementation. 

The effective use of information and communication technologies is necessary to optimize organizational processes influencing the improvement of the path of treatment of patients after THA, better access to health care services in this area, better treatment outcomes and improvement of the level of patient safety. We assume that after the implementation of our Project to the health care system in Poland, we will obtain similar results, which requires further research.

## 5. Conclusions

This paper presented an original process using contemporary telemedicine technologies and eliminates most of disadvantages of the current and recommended by AOTMiT posthospital patient care process in Poland after THA. 

The designed process:

1. Can increase the patient’s safety;
2. Enables the conduction of rehabilitation remotely in the event of the lack of access to outpatient physiotherapy services or the lack of the patient transport to the rehabilitation center;
3. It may contribute to shortening the convalescence time, reducing the risk of complications, as well as reducing treatment costs.

The process of care using modern telemedicine technologies will allow for regular, remote contact with specialists, the continuation of rehabilitation in home conditions and the monitoring of parameters related to activity and location when there is no possibility of personal contact with specialists, e.g., during a pandemic.

The designed process is ready for further research with doctors, nurses, physiotherapists, administrative staff of a medical facility, patients after arthroplasty and patient caregivers’ participation. Eventually, the process may become a medical procedure used in the care of patients after THA. 

We recognized the need to implement the designed process within 2 years of its creation, at least in the form of an experiment on a smaller group of patients as a preliminary study, and then, with necessary modifications, to be implemented in a large group of patients. If not implemented within the proposed period, an update of the process will be required due to the rapid development of innovative technologies.

The clinical verification of the designed process is substantiated in the conditions defined below by the authors of the publication:

1. Current process of post-hospital care process for patient after THA is not applying the full potential of available telemedicine technologies.
2. High quality post-hospital care to maintain the intended effect of recovery is needed, increasing the number of costly THA procedures.
3. The post-hospital period for patients undergoing THA is important, including the monitoring of fails that may be the cause of repeated need for surgery.
4. This type of procedure minimizes personal interaction a contributing to limiting epidemiological threats development, for example, those caused by the COVID-19 pandemic.

Introducing the process to clinical practice will allow to estimate the health and economic effects, taking into account the current costs of technologies used in the developed process and social perception, which after the COVID-19 pandemic is definitely more conducive to remote communication and monitoring.

## Figures and Tables

**Table 1:** Aspects of interest in participant observation.

Aspect of Interest/Subject of Observation	Observed Occupational Group	Number of Participant Observation
Types of rehabilitation in posthospital patient care process.	orthopedist, physiotherapist, nurse	4
Physiotherapists instructions directed to patient in hospital in the field of self-rehabilitation at home in posthospital period.	physiotherapist	4
Patient registration process for control visits in posthospital period.	reception personnel	4
Observation on video-consultation tool implemented as commercial service for patients.	orthopedist, administration personnel	2

## Data Availability

The data presented in this study are available on request from the corresponding author.

## Supplementary Materials

The following are available online at https://www.mdpi.com/article/10.3390/ijerph181910135/s1, Participant observation sheet, Structured interviews sheets.
