# Supplementary material for: Process of Posthospital Care Involving Telemedicine Solutions for Patients after Total Hip Arthroplasty
Source: Int J Environ Res Public Health. 2021 Sep 27;18(19):10135. doi: 10.3390/ijerph181910135 (PMC8508342; doi:10.3390/ijerph181910135)
Supplement: Supplementary file 1 [file ijerph-18-10135-s001.zip › ijerph-1273312-supplementary.pdf]

PARTICIPANT OBSERVATION SHEET

____
Sheet code

Participant observation during a process, activity or meeting

Date/week day	Place - address	Start time	End time	Duration

1. **List of activities under observation:**

- Subject of observation:
- Observation notes:

2. **Number of meeting participants by functions:**

3. **Remarks:**

Researcher's signature

INSTRUCTIONS FOR INTERVIEW

INTERVIEW WITH HEAD OF ORTOPEDIC SURGERY WARD UNIT________

Sheet code

Form of conversation [interview]: ☐ personal ☐ via telephone

Date/week day	Place - address	Start time	End time	Duration

1. **Characteristics of the intervieweas with role/function:**

- Person 1: ………
- Person 2: …………………………………………………………………… ……………………………………………………………

(e.g. patient's age, patient after hip arthroplasty 6 months / 1 year ago, reason for surgery)

2. **Planned topic/scope/questions to be interviewed:**

- Topic: ………
- Scope: ………
- Questions (Q):

Q1: How many standard follow-up visits are recommended by the ward director?

Q2: In what periods are the above-mentioned control visits?

Q3: What is the main goal of the next control visits?

Q4: Are additional control visits possible under the contract with the National Health Fund?

Q5: Do Patients receive recommendations for self-rehabilitation at home?

Q6: Do Patients receive a referral to outpatient rehabilitation, rehabilitation ward, orthopedic surgery clinic?

Q7: How does the patient obtain information on the postoperative wound hygiene and anticoagulant injections (is this an oral or written recommendation)?

Q8: Can a fall while walking damage the operated joint, if so, what are the consequences?

Q9: What is the cause of poor wound healing in the first week after surgery?

Q10: What is the cause of decreased joint mobility and when can it occur?

Q11: What is the cause of a joint dislocation?

Q12: Can the lack of rehabilitation be a cause of hip reoperation?

Q13: What are the causes of loosening or dislocation of the operated hip?

(topic of conversation or description of the scope and questions planned to be asked by the researcher's interlocutor)

3. **Respondent's answers (A)** *(open-ended answers):*

A1:……
……
……
A2:……
……
……
A3:……
……
……
A4:……
……
……
A5:……
……
……
A6:……
……
……
A7:……
……
……
A8:……
……
……
A9:……
……
……
A10:………
……
……
A11:………
……
……
A12:………
……
……
A13:………
……
……

4. Additional notes:

………

………

………

………

………

………

………

………

Researcher’s signature

INSTRUCTIONS FOR INTERVIEW

INTERVIEW WITH ORTOPEDIST WORKING AT OUTPATIENT SPECIALIST CARE UNIT________

Sheet code

Form of conversation [interview]: ☐ personal ☐ via telephone

Date/week day	Place - address	Start time	End time	Duration

5. **Characteristics of the intervieweas with role/function:**

- Person 1: ……
- Person 2: ………………………………………………………………………… ……………………………………………………………………

(e.g. patient's age, patient after hip arthroplasty 6 months / 1 year ago, reason for surgery)

6. **Planned topic/scope/questions to be interviewed:**

- Topic: ………
- Scope: ………
- Questions (Q):

Q1: How many standard follow-up visits are recommended by the head of the orthopedic surgery ward?

Q2: In what periods are the above-mentioned control visits?

Q3: What is the main goal of the next control visits?

Q4: Are additional control visits possible refunded by National Health Fund?

Q5: What is the cause of poor wound healing in the first week after surgery?

Q6: What is the cause of decreased joint mobility and when can it occur?

Q7: What is the cause of the operated hip dislocation?

Q8: Can a fall damage the operated joint, if so, what are the consequences?

(topic of conversation or description of the scope and questions planned to be asked by the researcher's interlocutor)

7. Respondent's answers (A) *(open-ended answers):*

A1:………
……
……
A2:………
……
……
A3:………
……
……
A4:………
……
……
A5:………
……
……
A6:………
……
……
A7:………
……
……
A8:………
……
……

8. Additional notes:

……
……
……
……
……
……
……
……

Researcher's signature

INSTRUCTIONS FOR INTERVIEW

INTERVIEW WITH NURSE WORKING AT THE TREATMENT ROOM OF OUTPAIENT SPECIALIST CARE_________

Sheet code

Form of conversation [interview]: ☐ personal ☐ via telephone

Date/week day	Date/week day	Date/week day	Date/week day	Date/week day

9. Characteristics of the intervieweas with role/function:

- Person 1: ………
- Person 2: ………

(e.g. patient's age, patient after hip arthroplasty 6 months / 1 year ago, reason for surgery)

10. Planned topic/scope/questions to be interviewed:

- Topic: ………
- Scope: ………
- Questions (Q):

Q1: How many follow-up visits are in the standard of care recommended by the head of the trauma and orthopedic surgery ward and the orthopedic physician of the orthopedic surgery clinic?

Q2: In what periods are the above-mentioned control visits?

Q3: What is the main purpose of the first follow-up visit?

(topic of conversation or description of the scope and questions planned to be asked by the researcher's interlocutor)

11. Respondent's answers (A) *(open-ended answers):*

A1:………

……

……

A2:………

……

……

A3:………

……

……

12. Additional notes:

………

………

………

………

………

………

………

………

Researcher’s signature

INSTRUCTIONS FOR INTERVIEW

INTERVIEW WITH AMBULATORY PHYSIOTHERAPY SUPERVISOR________

Sheet code

Form of conversation [interview]: ☐ personal ☐ via telephone

Date/week day	Place - address	Start time	End time	Duration

13. Characteristics of the intervieweas with role/function:

- Person 1: ………
- Person 2: ……………………………………………………………………………………… ……………………………………………………………………………

(e.g. patient's age, patient after hip arthroplasty 6 months / 1 year ago, reason for surgery)

14. Planned topic/scope/questions to be interviewed:

- Topic: ……
- Scope: ……
- Questions (Q):

Q1: What is the cause of decreased joint mobility and when can it occur?

Q2: What is the cause of the joint dislocation?

Q3: What is the cause of the pain in the scar after it healed?

Q4: In what form does the patient receive home rehabilitation recommendations?

Q5: How long does rehabilitation take after surgery?

Q6: What are the types of post-operative rehabilitation?

Q7: Do patients report that they have difficulties with transport and therefore give up rehabilitation?

Q8: Are the physiotherapists in the team familiar with telerehabilitation systems?

Q9: Are the physiotherapists on the Outpatient Physiotherapy team familiar with fall monitoring wearables?

Q10: Can a fall damage the operated joint, if so, what are the consequences?

(topic of conversation or description of the scope and questions planned to be asked by the researcher's interlocutor)

15. Respondent's answers (A) *(open-ended answers):*

A1:………

……

……

A2:………

……

....

A3:..

....

....

A4:..

....

....

A5:..

....

....

A6:..

....

....

A7:..

....

....

A8:..

....

....

A9:..

....

....

A10:..

....

....

16. Additional notes:

....

....

....

....

....

....

....

....

Researcher's signature

INSTRUCTIONS FOR INTERVIEW

INTERVIEW WITH SUPERVISOR OF THE OUTPATIENT SPECIALIST CARE PATIENT REGISTRATION DEPARTMENT________

Sheet code

Form of conversation [interview]: ☐ personal ☐ via telephone

Date/week day	Place - address	Start time	End time	Duration

17. Characteristics of the intervieweas with role/function:

- Person 1: ……
- Person 2: ……………………………………………………………………………… ……………………………………………………………………

(e.g. patient's age, patient after hip arthroplasty 6 months / 1 year ago, reason for surgery)

18. Planned topic/scope/questions to be interviewed:

- Topic: ………
- Scope: ………
- Questions (Q):

Q1: How many standard of care follow-up visits are recommended by the heard of orthopedic surgery hospital ward?

Q2: In what periods are the above-mentioned control visits?

Q3: What is the main goal of the next control visits?

Q4: Are additional or urgent control visits possible under the contract with the National Health Fund?

Q5: In what form is the patient informed about the follow-up appointments and is their appointment being reminded in some form to the patient by the upcoming appointment date?

Q6: Do Patients after hip arthroplasty ask about the orthopedic telepathic service after surgery?

Q7: During the pandemic period, did Patients cancel their follow-up visits, postponed or interchangeable telepaths?

(topic of conversation or description of the scope and questions planned to be asked by the researcher's interlocutor)

19. Respondent's answers (A) *(open-ended answers):*

A1:……

………

………

A2:……

………

………

A3:……
………
………
A4:……
………
………
A5:……
………
………
A6:……
………
………
A7:……
………
………

20. Additional notes:

………
………
………
………
………
………
………
………

Researcher’s signature

INSTRUCTIONS FOR INTERVIEW

INTERVIEW WITH THE OPERATOR
OF MEDICAL DEVICES STORE________

Sheet code

Form of conversation [interview]: ☐ personal ☐ via telephone

Date/week day	Place - address	Start time	End time	Duration

21. Characteristics of the intervieweas with role/function:

- Person 1: ……………………………………………………………………………………………………
- Person 2: ……………………………………………………………… ……………………………………………………

(e.g. patient's age, patient after hip arthroplasty 6 months / 1 year ago, reason for surgery)

22. Planned topic/scope/questions to be interviewed:

- Topic: ……………………………………………………………………………………………………
- Scope: ……………………………………………………………………………………………………
- Questions (Q):

Q1: Do patients after the procedure ask about wearable technologies in a hospital orthopedic supply store?

(topic of conversation or description of the scope and questions planned to be asked by the researcher's interlocutor)

23. Respondent's answers (A) *(open-ended answers):*

A1:……………………………………………………………………………………………………

……………………………………………………………………………………………………

……………………………………………………………………………………………………

24. Additional notes::

……………………………………………………………………………………………………

……………………………………………………………………………………………………

……………………………………………………………………………………………………

……………………………………………………………………………………………………

Researcher's signature

INSTRUCTIONS FOR INTERVIEW

INTERVIEW WITH THE PATIENT________

Sheet code

Form of conversation [interview]: ☐ personal ☐ via telephone

Date/week day	Place - address	Start time	End time	Duration

25. Characteristics of the intervieweas with role/function:

- Person 1: ……
- Person 2: …………………………………………………………………………………… ……………………………………………………………………………

(e.g. patient's age, patient after hip arthroplasty 6 months / 1 year ago, reason for surgery)

26. Planned topic/scope/questions to be interviewed:

- Topic: ………
- Scope: ………
- Questions (Q):

Q1: How many follow-up visits at the outpatient specialist care did you have after hip arthroplasty within a year after the surgery?

Q2: What forms of rehabilitation did you use in the first year after the surgery?

Q3: How long did the wound take to heal and were there any problems with caring for it?

Q4: Did you fall in the first year after the surgery, if so, were you alone at that time?

Q5: When did you stop using your elbow crutches after the surgery?

Q6: How long have you been achieving the expected efficiency?

Q7: Have you heard about fall-alarming wristbands and would you decide to use them after the procedure?

Q8: Do you use Nationl Health Fund teleconsultations or commercial teleconsultations?

Q9: How long was the hospital stay after surgery?

Q10: Did you live with your guardian after the surgery? If so, for how long after the procedure?

Q11: Have you provided yourself with additional products, such as a wedge, a toilet seat or other?

Q12: What do you remember from the first year after the procedure?

Q13: Were there any complications after the procedure, and if so what?

Q14: Have you exercised yourself at home after surgery within a year of surgery?

(topic of conversation or description of the scope and questions planned to be asked by the researcher's interlocutor)

27. Respondent's answers (A) *(open-ended answers):*

A1:……

………

………

A2:……

………

………

A3:……

………

………

A4:……

………

………

A5:……

………

………

A6:……

………

………

A7:……

………

………

A8:……

………

………

A9:……

………

………

A10:………

………

………

A11:………

………

………

A12:………

………

………

A13:………

………

………

A14:………

………

………

4. Additional notes:

………

………

………

………

………

………

………

………

Researcher’s signature
